# A Novel Dual‐Task Paradigm for Return‐to‐Sport Screening After ACL Injury: A Pilot Study

**DOI:** 10.1155/tsm2/1073180

**Published:** 2026-01-09

**Authors:** Alva Lövgren, Andrew Strong, Carl-Johan Boraxbekk, Jonas L. Markström

**Affiliations:** ^1^ Department of Community Medicine and Rehabilitation, Unit of Physiotherapy, Umeå University, Umeå, Sweden, umu.se; ^2^ Institute for Clinical Medicine, Faculty of Medical and Health Sciences, University of Copenhagen, Copenhagen, Denmark, ku.dk; ^3^ Institute of Sports Medicine Copenhagen (ISMC), Copenhagen University Hospital, Bispebjerg, Copenhagen, Denmark, gentoftehospital.dk; ^4^ Department of Neurology, Copenhagen University Hospital, Bispebjerg, Copenhagen, Denmark, gentoftehospital.dk

**Keywords:** cognition, dual-task, knee, ligaments, rehabilitation, test–retest

## Abstract

**Background:**

Current return‐to‐sport screening paradigms after anterior cruciate ligament (ACL) injury are inadequate as they fail to reflect cognitive‐motor sports demands. This pilot study aimed to evaluate dual‐task ability in individuals with ACL reconstruction (ACLR) using a novel dual‐task test paradigm. Specifically, we compared (1) cognitive and motor performance between individuals with ACLR and controls, (2) hop test performance between the injured and non‐injured legs within the ACLR group, and (3) performance across test‐retest sessions.

**Materials and Methods:**

Twenty sports active individuals (10 ACLR, 10 controls) performed the dual‐task paradigm twice within a week, comprising a cognitive test, a dual‐task drop‐vertical hop test, and an upper‐body hand‐tapping test. All tests incorporated a visuospatial working‐memory task (cognitive performance), with the latter two additionally engaging attention, decision‐making, and inhibitory control (motor performance). Between‐group, between‐leg, and test‐retest differences were analyzed using independent and paired *t*‐tests with Cohen’s d effect sizes (ESs). Test–retest reliability was examined using intraclass correlation coefficient (ICC), along with the within‐person standard deviation and minimal detectable change.

**Results:**

No significant differences were observed between ACLR and controls at the first test session (*p* = 0.09 − 0.34; ESs = 0.19–0.62 [very small–medium]), although ACLR mean performances were 3.8%–14.1% lower. At retest, ACLR performed significantly worse than CTRL for most outcomes (*p* = 0.01 − 0.03; ESs = 0.91–1.17 [large]) and showed smaller improvements for a hop test outcome (*p* = 0.04; ES = 0.97 [large]). No differences were found between ACLR legs, both groups improved across test sessions, and test–retest reliability was excellent for ACLR (ICCs = 0.74–0.97) and ranged from poor to excellent in CTRL (ICCs = 0.19–0.86).

**Conclusions:**

This pilot study demonstrates the feasibility and preliminary reliability of the dual‐task paradigm, particularly within the ACLR group. Poorer cognitive, hop, and upper‐body test performances and smaller test–retest improvements for the ACLR group suggest persistent dual‐task deficits following injury, supporting the paradigm’s utility for ecologically valid ACL rehabilitation and return‐to‐sport assessment.

## 1. Introduction

Most anterior cruciate ligament (ACL) injuries and reinjuries occur without direct contact, typically during high‐velocity cutting or landing when attention is divided [1, 2]. Despite this, screening tests for return to sport (RTS) after ACL injury usually assess only thigh muscle strength, knee range of motion, and hopping ability, often using the noninjured leg as a reference [3]. Determining whether such asymmetries result from the injury itself or predate it requires data collected both before and after injury, which are rarely available. Even after physiotherapist‐led rehabilitation usually lasting a year, many individuals fail to RTS due to poor perceived knee function and fear [4], while 20%–30% of those who do return sustain a reinjury [5, 6]. These high reinjury rates and the mismatch between real‐world injury mechanisms and current assessments highlight the need to incorporate simultaneous cognitive and motor demands into rehabilitation and screening [7–9].

Dual‐task scenarios can lead to performance deficits in one or both tasks, a phenomenon known as cognitive‐motor interference or the dual‐task cost [10]. Individuals with previous ACL injury show greater dual‐task costs across a range of motor tasks. Ness and colleagues [11] highlighted in their review that studies adding cognitive load during single‐limb stance and gait revealed both motor and cognitive performance costs in those with ACL injury compared to healthy individuals. Strong et al. [12] extended these findings to a drop vertical jump with secondary cognitive tasks. These studies show that dual‐task deficits are evident in both everyday and high‐impact movements for individuals with ACL injury. These impairments may reflect the loss of ligament sensory receptors [13] and subsequent central nervous system adaptations [14].

However, it remains unclear whether ACL injury causes impaired dual‐task ability or whether preexisting deficits increase injury risk. If the latter is true, such deficits may affect whole‐body movement, including both lower limbs and upper‐body function. Previous studies involving high‐impact cognitive‐motor dual‐tasking have mainly used single‐leg hops, tested only in healthy participants, and paired with simple cognitive tasks such as letter or color recognition [12, 15–17]. To address these limitations, the present study used a novel and more demanding dual‐task paradigm that integrates complex cognitive processing with both lower‐ and upper‐body motor tasks, enabling a more comprehensive assessment of dual‐task ability in individuals with ACL injury. Such tools may allow clinicians to better evaluate dual‐task performance after ACL injury, to inform RTS decisions, and potentially to reduce reinjury risk.

This pilot study aimed to evaluate dual‐task ability in individuals with and without ACL injury using a novel dual‐task paradigm comprising three tests: a cognitive test (visuospatial working memory), a dual‐task hop test integrating the same cognitive task with a single‐leg hop landing task requiring attention, rapid decision‐making, and inhibitory control, and an upper‐body control test applying the same dual‐task principles to hand movements. Specifically, we compared (1) cognitive and motor performance between groups (2), hop test performance between the injured and noninjured legs within the ACL group, and (3) performance between test and retest sessions for each group.

## 2. Materials and Methods

### 2.1. Participants

The test–retest pilot study involved 20 sports‐active individuals, 10 (3 males) with ACL reconstruction (ACLR) and 10 (4 males) noninjured controls (CTRL) (Table 1). Two participants with ACLR were enrolled but later excluded for not completing the tests, one due to apprehension regarding the unanticipated nature of the hop test during the first test session (despite having returned to floorball at a competitive level) and the other due to overall knee discomfort with the hop test during the retest session. These two individuals were replaced, resulting in the 20 participants completing all tests for both test sessions. Given the exploratory nature of this pilot study, no formal sample‐size calculation was performed. The sample was deemed sufficient to address the study aims and to inform future large‐scale validation of the novel paradigm. Inclusion criteria were as follows: a minimum of 15 years of age, a Tegner activity scale [18] rating ≥ 6/10, RTS containing unpredictable and rapid directional changes performed weekly, confident in performing maximal hop and strength tests, unilateral ACL injury, ipsilateral hamstring graft (standard national practice), no concomitant injuries, no severe ankle sprain within six months, no other pathology that would affect their ability to hop, no color blindness. Controls were required to meet the same criteria, excluding ACL injury, and without recent or functionally significant lower limb injuries. All participants gave written informed consent to participate, and the National Ethical Review Authority (Dnr. 2024‐05292‐02) approved the research procedures.

**Table 1:** Participant demographics.

	**ACLR**	**CTRL**

Male/female, *n*	3/7	4/6
Age, years, mean (SD)	24.3 (3.4)	26.0 (5.6)
Body mass index, kg/m^2^, mean (SD)	24.9 (2.1)	24.5 (2.4)
Time since ACLR, months, mean (min–max)	39.9 (21.5–55.4)	—
Tegner rating, score, mean (SD)	7.8 (1.4)	8.5 (1.1)

*Note:* ACLR, anterior cruciate ligament reconstruction group; CTRL, control group.

Abbreviation: SD, standard deviation.

### 2.2. Study Procedures

Participants wore their own sports clothing and completed the cognitive test, followed by the dual‐task hop test, and then the dual‐task upper‐body test (see Figure 1 for details; the complete protocol can be provided upon request). The cognitive test targeted working memory with an emphasis on visuospatial stimuli over 16 repetitions. The hop test combined this test with a hop task requiring attention, fast decision‐making, and inhibitory control across 32 repetitions, divided into two 16‐trial sets. Each set included two blue and two green arrows pointing up and down per leg, separated by a five‐min rest. The number of trials was deemed sufficient to ensure reliable comparisons between groups and legs within the ACLR group without inducing fatigue that could confound performance or observing ceiling effects. The upper‐body test mirrored the hop test but replaced hopping with an upper‐body hand‐tapping task, performed over 16 repetitions, which was considered suitable since the test was administered last, when mental fatigue could begin to emerge. The hop test was preceded by a standardized warm‐up involving squats, squat jumps, heel raises, and single‐leg drop landings. Test‐specific practice trials were performed before each test: two for the cognitive test, eight for the motor‐only condition and two dual‐task trials for the hop test, and eight for the motor‐only condition for the upper‐body test. Each participant repeated the test session within a week (on average 4.2 [SD 1.3] days after) to avoid unrelated disturbances affecting performance. Identical verbal and visual instructions were provided by the same assessor (JLM) in both sessions. Visual stimuli were presented via PowerPoint with timing controlled by its built‐in functions, while the assessor (JLM) manually advanced the arrows using a wireless presenter (Logitech, R400).

Figure 1The cognitive test (a). Participants memorized four figures presented for 5 s in a 2 × 2 matrix on a TV screen. Each figure combined three partially overlapping geometric shapes in four or five colors, with most colors appearing at least twice to prevent reliance on solely shape or color cues. After a 5‐s countdown, one figure from the set was shown for 0.5 s, followed by a 3‐s delay. All four figures then reappeared in random order in a horizontal row with a “None” option. Participants indicated which figure had been initially positioned above, below, to the right, or to the left of the briefly shown figure (element of working memory). Because “None” was correct for two sides of each figure, it appeared less often as the correct answer than the other options. This test involves 16 trials. The dual‐task hop test (b). Standing on a 35‐cm platform, participants viewed the four figures for 5 s, followed by the 5‐s countdown. At drop initiation, a blue or green arrow pointing upward or downward appeared for 0.5 s on either side of the screen, with one of the memorized figures inside (elements of attention and fast decision‐making). The arrow’s screen position indicated the landing leg. A downward green arrow signaled “land only” and an upward green arrow signaled “land and immediately hop forward.” Blue arrows required the opposite action to green arrows (element of inhibitory control). Participants were required to note the figure, perform the correct leg action, and then answer the working‐memory question. This test involves 32 trials, divided into two 16‐trial sets separated by a five‐minute rest (= two blue and two green arrows pointing up and down per leg per set). The dual‐task upper‐body test (c). Seated at a table, participants faced a 2 × 2 matrix of four 10 × 10 cm textile squares (cut from a mouse pad) spaced 25 cm apart. Starting with hands above the two proximal squares, they responded to arrow cues containing one of the memorized figures. The arrow direction indicated the horizontal position (left/right) and the vertical position (up = distal, down = proximal) of the target square. Blue arrows required tapping the square with the opposite vertical position. Participants were required to note the figure, tap the correct square as quickly as possible, and then answer the working‐memory question. This test involves 16 trials (= two blue and two green arrows pointing up and down per arm).

**Figure:**
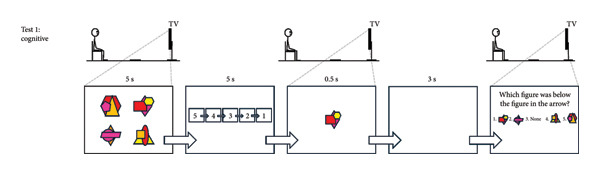
(a)

**Figure:**
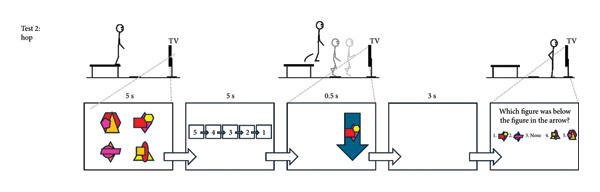
(b)

**Figure:**
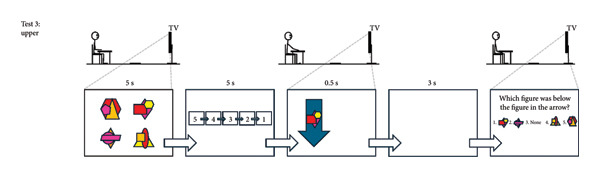
(c)

### 2.3. Outcome Variables

Cognitive and motor performances were selected as primary outcomes because they capture the key components of dual‐task ability targeted by the novel paradigm, which integrates cognitive processing with sport‐relevant motor tasks that seem affected by ACL injury [11, 12, 14]. The three tests were administered in a fixed sequence to promote comparable starting conditions for the more demanding hop and upper‐body dual‐task tests for participants. The cognitive test, conducted first, assessed performance as the percentage of correct responses across the 16 trials. In the hop test, performed second, a landing was deemed correct if the participant maintained balance for at least two seconds without foot shuffling or contralateral foot contact. For the landing‐and‐hop‐forward condition, the action was correct if the participant landed, immediately hopped forward at least half a meter without noticeable pause, and landed on the same leg without contralateral foot contact. Two assessors (JLM, AL) developed the paradigm together and performed training sessions to achieve consensus on the rating criteria. The two assessors then independently rated motor performance on separate protocols, and results were reported as the percentage of correct trials out of 32. Cognitive performance was also calculated across these trials. Combined cognitive‐motor success required both ratings to be correct, presented as a percentage of the 32 trials. Finally, in the upper‐body test, motion performance was scored as correct if participants tapped the correct square within approximately one second. The two assessors again independently rated motor performance, reported as the percentage of correct trials out of 16. Cognitive performance was calculated similarly, and combined success required both cognitive and motor criteria, reported as a percentage of the 16 trials. The assessors compared their results for agreement after each test session. Discrepancies in a few trials (out of 960 total per test session from the jump and upper‐body tests [48 trials ∗ 20 individuals]) were resolved through discussion, resulting in full consensus. Due to near‐perfect agreement, formal inter‐rater reliability statistics were not calculated.

### 2.4. Statistical Analyses

Independent *t*‐tests were conducted to compare cognitive and motor performance between the ACLR and CTRL groups (Aim 1). Within the ACLR group, paired *t*‐tests were used to evaluate differences in hop test performance between the injured and noninjured legs (Aim 2). Paired *t*‐tests were also used to assess test–retest differences in performance within each group (Aim 3). The test–retest reliability of the outcome measures was further examined using intraclass correlation coefficients (ICCs), calculated with the ICC (3, k) model (two‐way mixed‐effects, consistency, average measures), and interpreted according to Fleiss (ICC < 0.4 = poor, ICC 0.4–0.75 = fair to good, ICC > 0.75 = excellent) [19]. Bland–Altman plots were used to assess systematic bias, outliers, and heteroscedasticity in the test–retest data. Agreement was further evaluated using the within‐person standard deviation (*S*_*W*_), calculated as the square root of the average within‐subject variances, providing clinically relevant estimates of expected individual measurement error [20]. Minimal detectable change (MDC) for each variable was also calculated by constructing a 95% confidence interval around *S*_*W*_, accounting for variability across both test sessions.

Given the small sample size, statistical results should be interpreted with caution, as the study was designed to identify preliminary trends rather than to draw confirmatory conclusions. Therefore, Cohen’s d effect sizes (ESs, < 0.2 = very small, 0.2 = small, 0.5 = medium, 0.8 = large) [21] were also reported for all *t*‐tests to indicate the magnitude of observed differences. All analyses were conducted in SPSS (v.23, IBM, Armonk, New York, USA) with a 5% significance level set *a priori*.

## 3. Results

### 3.1. Differences Between Groups

In the first test session, no statistically significant differences were observed between the groups for any outcome (*p* = 0.09 − 0.34). Mean performances were 3.8%–14.1% lower for ACLR compared to CTRL, with medium ESs for the cognitive and all three upper‐body outcomes. At retest, ACLR demonstrated statistically significant poorer performance than CTRL for four outcomes (cognitive, hop motor, upper‐body cognitive, and upper‐body combined; *p* = 0.01 − 0.03; ESs = 0.91–1.17 [large]), while the remaining did not statistically differ (hop cognitive, hop combined, and upper‐body motor; *p* = 0.06 − 0.36; ESs = 0.17–0.72 [very–small to medium]). ACLR also revealed smaller test–retest improvements than CTRL for hop motor (*p* = 0.04; ES = 0.97 [large]), but not for the remaining outcomes (*p* = 0.19 − 0.66; ESs = −0.20 to 0.61 [small to medium]) (Table 2).

**Table 2:** Results for between‐group comparisons, presented as correct performances in percentage out of 16 trials for the cognitive and upper‐body tests and 32 trials for the hop test.

	**Test**	**ACLR**	**CTRL**	**ACLR-CTRL**		
		**Mean (SD)**	**Mean (SD)**	**MD (SE)**	**Cohen’s d**	**p** **value**

*Cognitive test*						
Cognitive performance	Test 1	60.7 (20.6)	70.0 (12.8)	9.3 (7.7)	0.54 (M)	0.12
	Test 2	77.7 (20.8)	93.3 (7.5)	15.6 (7.0)	1.00 (L)	**0.02**
	Diff T2‐T1	17.0 (18.2)^∗^	23.3 (11.0)^∗^	−6.3 (6.7)	0.42 (S)	0.36

*Hop test*						
Motor performance	Test 1	62.2 (10.6)	67.9 (13.5)	5.7 (5.4)	0.47 (S)	0.15
	Test 2	63.6 (11.4)	77.5 (12.4)	13.9 (5.3)	1.17 (L)	**0.01**
	Diff T2‐T1	1.4 (7.7)	9.6 (9.1)^∗^	−8.2 (3.8)	0.97 (L)	**0.04**

Cognitive performance	Test 1	54.5 (19.9)	61.3 (21.2)	6.8 (9.2)	0.33 (S)	0.23
	Test 2	70.9 (23.6)	74.1 (14.0)	3.2 (8.7)	0.17 (VS)	0.36
	Diff T2‐T1	16.4 (7.8)^∗^	12.8 (24.1)	3.6 (8.0)	−0.20 (S)	0.66

Motor + cognitive performance	Test 1	40.7 (18.3)	44.5 (21.2)	3.8 (8.8)	0.19 (VS)	0.34
	Test 2	46.6 (20.7)	59.9 (15.8)	13.3 (8.2)	0.72 (M)	0.06
	Diff T2‐T1	5.9 (9.4)^∗^	15.4 (20.1)^∗^	−9.5 (7.0)	0.61 (M)	0.19

*Upper-body test*						
Motor performance	Test 1	83.3 (9.9)	88.9 (10.6)	5.6 (4.6)	0.55 (M)	0.12
	Test 2	90.8 (6.8)	94.6 (3.4)	3.8 (2.4)	0.71 (M)	0.07
	Diff T2‐T1	7.5 (7.7)^∗^	5.7 (10.0)	1.8 (4.0)	−0.20 (S)	0.66

Cognitive performance	Test 1	60.1 (25.7)	73.2 (19.1)	13.1 (10.1)	0.58 (M)	0.11
	Test 2	68.3 (28.7)	88.3 (9.5)	20.0 (9.6)	0.93 (L)	**0.03**
	Diff T2‐T1	8.2 (11.4)^∗^	15.1 (13.7)^∗^	−6.9 (5.6)	0.55 (M)	0.24

Motor + cognitive performance	Test 1	51.9 (23.8)	66.0 (21.2)	14.1 (10.1)	0.62 (M)	0.09
	Test 2	63.2 (29.6)	83.3 (9.4)	20.1 (9.8)	0.91 (L)	**0.03**
	Diff T2‐T1	11.3 (12.1)^∗^	17.3 (15.8)^∗^	−6.0 (6.3)	0.43 (S)	0.35

*Note:* ACLR, anterior cruciate ligament reconstruction group; CTRL, control group; Cohen’s d is classified as very small (VS), small (S), medium (M), and large (L). Statistically significant differences between groups are highlighted with *p* values in bold.

Abbreviations: MD, mean differences; SD, standard deviation; SE, standard error.

^∗^Statistically significant difference between test sessions within groups (*p* < 0.05).

### 3.2. Differences Between Legs of ACLR

No significant differences were found between the injured and noninjured legs in cognitive, motor, or combined performance during the hop test at either test session (*p* = 0.60 − 1.0; ESs = 0.00–0.17 [very small]). The average performance of the injured leg was 0%–3.1% better than for the noninjured leg (Table 3).

**Table 3:** Results for between‐leg comparisons for the hop test within the ACLR group, presented as correct performances in percentage out of 16 trials.

	**Test**	**I-leg**	**NI-leg**	**Difference I-leg—NI-leg**		
		**Mean (SD)**	**Mean (SD)**	**Mean (95% CI)**	**Cohen’s d**	**p** **-value**

*Hop test*						
Motor performance	Test 1	62.5 (14.7)	61.9 (9.5)	0.6 (−8.7–9.9)	0.05 (VS)	0.88
	Test 2	63.8 (16.1)	63.1 (10.0)	0.6 (−9.1–10.4)	0.00 (VS)	0.89

Cognitive performance	Test 1	54.4 (24.8)	54.4 (18.6)	0.0 (−13.4–13.3)	0.15 (VS)	1.0
	Test 2	73.1 (24.7)	70.0 (21.6)	3.1 (−9.9–16.1)	0.05 (VS)	0.60

Motor + cognitive performance	Test 1	41.9 (22.3)	39.4 (17.7)	2.5 (−9.7–14.6)	0.17 (VS)	0.65
	Test 2	46.9 (27.0)	46.3 (15.6)	0.6 (−11.0–12.2)	0.04 (VS)	0.91

*Note:* ACL‐injured leg; NI‐leg, noninjured leg.

Abbreviations: CI, confidence interval; SD, standard deviation; VS, very small.

### 3.3. Differences Across Test Sessions

Both groups demonstrated performance improvements from test to retest, although the magnitude and consistency varied across outcomes. For ACLR, significant improvements were observed for cognitive (*p* = 0.02; ES = 0.94 [large]), hop cognitive (*p* < 0.01; ES = 2.09 [large]), upper‐body motor (*p* = 0.01; ES = 0.96 [large]), upper‐body cognitive (*p* = 0.05; ES = 0.72 [medium]), and upper‐body combined (*p* = 0.02; ES = 0.94 [large]), but not for hop motor (*p* = 0.58; ES = 0.18 [very small]) or hop combined (*p* = 0.08; ES = 0.63 [medium]). For CTRL, improvements were significant for cognitive (*p* < 0.01; ES = 2.12 [large]), hop motor (*p* = 0.01; ES = 1.05 [large]), hop combined (*p* = 0.04; ES = 0.77 [medium]), upper‐body cognitive (*p* = 0.01; ES = 1.10 [large]), and upper‐body combined (*p* = 0.01; ES = 1.10 [large]), but not for hop cognitive (*p* = 0.13; ES = 0.53 [medium]) or upper‐body motor (*p* = 0.11; ES = 0.57 [medium]). Individual‐level paired test–retest data are presented in Figure S1 in the Supporting Information.

Regarding reliability, ACLR showed excellent test–retest reliability across all outcomes (ICCs = 0.76–0.97) except upper‐body motor (ICC = 0.74, good), with S_Ws_ ranging from 5.4% to 12.8% and MDs ranging from 15.0% to 35.6%. CTRL showed excellent reliability for hop motor (ICC = 0.86), fair‐to‐good reliability for cognitive, hop combined, upper‐body cognitive, and upper‐body combined (ICCs = 0.60–0.74), and poor reliability for hop cognitive (ICC = 0.19) and upper‐body motor (ICC = 0.31), with S_Ws_ ranging from 6.4% to 17.0% and MDCs ranging from 17.8% to 47.2% (Table 4). Bland–Altman plots for each outcome are presented in Figure S2 in the Supporting Information.

**Table 4:** Test–retest reliability across sessions for each outcome, presented with intraclass correlation coefficient (ICC), with average within‐person standard deviations (*S*_*W*_) and minimal detectable changes (MDC) reported as percentages.

	**ACLR**			**CTRL**		
	**ICC**	**S**_**W**_^**∗**^	**MDC** ^**∗**^	**ICC**	**S**_**W**_^**∗**^	**MDC** ^**∗**^

*Cognitive test*						
Cognitive performance	0.76	12.8	35.6	0.62	7.8	21.6

*Hop test*						
Motor performance	0.86	5.4	15.0	0.86	6.4	17.8
Cognitive performance	0.97	5.5	15.4	0.19	17.0	47.2
Motor + cognitive performance	0.94	6.6	18.4	0.60	14.2	39.3

*Upper-body test*						
Motor performance	0.74	5.4	15.1	0.31	7.1	19.6
Cognitive performance	0.95	8.1	22.3	0.74	9.7	26.9
Motor + cognitive performance	0.95	8.5	23.6	0.70	11.2	30.9

*Note:* ACLR, anterior cruciate ligament reconstruction group; CTRL, control group.

## 4. Discussion

This is the first study to employ a test paradigm that incorporates a reference test and an upper‐body control test, in addition to a high‐impact hop test, to assess dual‐task ability in individuals with ACLR. Across all three tests, individuals with ACLR generally demonstrated poorer cognitive, motor, and combined performance, along with generally smaller test–retest improvements, compared to CTRL. Our results for the hop test are consistent with previous findings of poorer cognitive‐motor performance during bilateral drop‐vertical jumps among individuals with ACLR who had achieved RTS [12]. Similarly, deficits in neurocognitive domains such as response speed, sustained attention, working memory, cognitive flexibility, and response inhibition have been reported in ACLR populations using CANTAB [22]. This combined evidence indicates that individuals with ACLR underperform across a broad range of cognitively demanding tests, from purely cognitive assessments to integrated cognitive‐motor dual‐tasking challenges involving both lower and upper‐body control.

It is unclear whether cognitive‐motor deficits represent a preexisting risk factor, a consequence of the injury, or a combination of both. In support of preexisting deficits, a prospective study shows that athletes who later sustain an ACL injury already perform worse on traditional neuropsychological tests compared to those who stay injury‐free [23]. Similarly, concussed athletes—who often show deficits in neuropsychological characteristics—have a higher overall injury risk than those without a concussion history [24, 25]. Poor cognitive performance has also been linked to biomechanical patterns considered associated with increased ACL injury risk in noninjured individuals [26]. However, in support of the injury being a causal factor, the loss of sensory receptors found in the ACL following rupture [13] may reduce brain connectivity [27] and trigger central nervous system adaptations [14, 28]. Furthermore, neuroimaging studies have identified atypical brain responses in ACL‐injured individuals during isolated lower limb movements [29–31], though not during proprioception tasks [32]. Such neural adaptations may compromise dual‐task ability, potentially increasing reinjury risk [7, 33]. Taken together, previous prospective research and cross‐sectional research on individuals with ACL injury, the latter complemented by the present findings, suggest that both preexisting deficits and injury‐related neural adaptations may contribute to impaired dual‐task performance, potentially increasing risk of reinjury. Future prospective studies should investigate whether deficits in cognitive‐motor dual‐task performance are predictive of increased injury risk.

The poorer overall performances among ACLR compared to CTRL may reflect a limitation in information processing capacity [34], whereas the smaller retest improvements likely reflect slower familiarization with the dual‐task demands rather than true gains in cognitive or motor capacity. This limitation may require participants to allocate greater cognitive resources to the cognitive element of the dual‐task, which could negatively impact motor task performance or vice versa, depending on task prioritization. Such findings align with recommendations that both injury prevention [35, 36] and rehabilitation strategies [7, 8, 33] incorporate cognitive‐motor dual‐task training to better prepare athletes for sports‐specific demands. Notably, despite all our participants having achieved RTS, one declined the hop test at the first test session due to apprehension regarding the unanticipated nature, and another declined at retest due to overall knee discomfort. These cases indicate that the hop test may be particularly relevant for late‐stage rehabilitation and RTS screening to identify those who may require further intervention.

Our dual‐task hop test presented a suitably high challenge, with group means for the combined cognitive and motor performance of 41%–45% correct trials at baseline and 47%–60% at retest (Table 2). This challenge point avoids ceiling and floor effects, allowing measurable improvement while avoiding a potential loss of motivation that could cause participants to stop trying [37]. The present findings support the feasibility of the proposed dual‐task paradigm and indicate preliminary reliability across sessions, particularly within the ACLR group reporting ICCs of 0.74–0.97 (Table 4). The observed between‐group differences, particularly apparent at retest, also suggest behavioral sensitivity and highlight the paradigm’s potential for broader validation and clinical application in RTS assessment. However, these findings should be interpreted alongside the ICCs among CTRL, which indicate that motor and combined performances, but not the cognitive performance, were reliable during the hop test. In contrast, the motor task in the upper‐body test was less demanding, as it lacked a similar component of urgency and time pressure compared to the hop test’s requirement for pre‐impact action. This resulted in higher performance success rates and smaller SDs in both groups, and consequently lower ICCs despite relatively small *S*_*W*_. If an upper‐body dual‐task test is intended to serve as a control for a lower‐body task such as the hop test, it should incorporate a motor task with strict time constraints and immediate feedback to better simulate the rapid decision‐making demands of sport.

### 4.1. Strengths and Limitations

This pilot study is an initial step in developing a dual‐task paradigm for injury and reinjury risk assessment, with future work focusing on refinement and larger samples. The main limitation is the small sample size (*n* = 10 per group), reducing statistical power. The ESs indicate the magnitude of observed differences and should be considered together with the statistical analysis to identify trends for future research rather than to draw confirmatory conclusions. Other limitations include uncontrolled factors in the ACLR group (e.g., rehabilitation protocols, surgical details), a lack of motor‐only baseline for the hop and upper‐body tests (not included due to potential fatigue), restricting us from calculating a true dual‐task cost, and the inclusion of both males and females, given potential sex‐related effects. However, all participants were carefully screened to meet the inclusion criteria, and Strong et al. [12] found no sex differences in dual‐task ability. Although a fixed test order was applied to maintain procedural consistency, this may have introduced order effects related to fatigue or task familiarity, which should be considered when interpreting the results. Lastly, the observed test–retest improvements suggest learning effects, which may have decreased ICCs and inflated the MDCs (particularly among CTRL). Future studies should include familiarity sessions, which were not used in the present study, to minimize learning effects.

Key strengths include the use of three distinct, literature‐informed and pilot‐tested tasks, designed to minimize ceiling effects (e.g., combining shapes and colors in the cognitive task to reduce reliance on mnemonic strategies) and (particularly the hop task) to reflect the demands in chaotic sports and recreational activities (e.g., ball sports) where ACL injuries are common [7–9]. The high‐impact motor task was chosen because it is considered suitable for RTS testing, where the athlete should be capable of controlling unplanned high‐impact landings while attending to external stimuli. The upper‐body test also allows for dual‐task assessment early after ACLR, before athletes can safely perform high‐impact motor tasks. The paradigm is also practical, requiring only one assessor, a platform for jumping, and a laptop with basic software, making it feasible for most clinical and sports settings. However, further research with larger sample sizes is needed to verify our findings and establish evidence‐based cutoff values for acceptable performance, preferably using dual‐task testing, since traditional assessments of neuropsychological testing (pen and paper, digital) or motor function do not adequately reflect cognitive‐motor test performance [38].

## 5. Conclusions

The present pilot study demonstrates the feasibility of the proposed dual‐task paradigm and provides preliminary evidence of reliability across test sessions, particularly within the ACLR group. Observed poorer cognitive, hop, and upper‐body test performances among individuals with ACLR compared to CTRL, especially at retest, as well as smaller test–retest improvements, suggest the paradigm is sensitive to behavioral performance, supporting its potential utility in assessing dual‐task ability relevant to RTS decisions. However, no differences emerged between the injured and noninjured legs of the ACLR group for the hop test. Overall, this novel dual‐task paradigm offers a clinically applicable method to improve the ecological validity of RTS testing, encouraging further research to refine and expand dual‐task assessments in ACL rehabilitation and (re)injury prevention.

## Conflicts of Interest

The authors declare no conflicts of interest.

## Funding

This study was funded by Umeå University Strategic Research Area Health Care Science, SFO‐V, and the Novo Nordisk Foundation grant to Team Danmark.

## Supporting Information

Additional supporting information can be found online in the Supporting Information section.

## Supporting information


**Supporting Information 1** Supporting 1: Figure S1. Individual paired data for the cognitive (= cog) and motor performances between tests for the cognitive test (= Cognitive), hop test (= Hop), and upper‐body test (= Upper) for each group. ACLR, anterior cruciate ligament reconstruction group; CTRL, control group.

**Supporting Information 2** Supporting 2: Figure S2. Bland–Altman plots for all outcomes. The *X* and *Y* axes have identical ranges to allow immediate visual comparisons between them. Identical values are stacked, so only the topmost dot is shown where present.

## Data Availability

The data that support the findings of this study are available from the corresponding author upon reasonable request.
