# Supplementary material for: A Novel Dual‐Task Paradigm for Return‐to‐Sport Screening After ACL Injury: A Pilot Study
Source: Transl Sports Med. 2026 Jan 9;2026:1073180. doi: 10.1155/tsm2/1073180 (PMC12788982; doi:10.1155/tsm2/1073180)
Supplement: Supplementary file 1 — Supporting Information 1 Supporting 1: Figure S1. Individual paired data for the cognitive (= cog) and motor performances between tests for the cognitive test (= Cognitive), hop test (= Hop), and upper‐body test (= Upper) for each group. ACLR, anterior cruciate ligament reconstruction group; CTRL, control group. [file TSM2-2026-1073180-s002.docx]

**SUPPLEMENT 1**


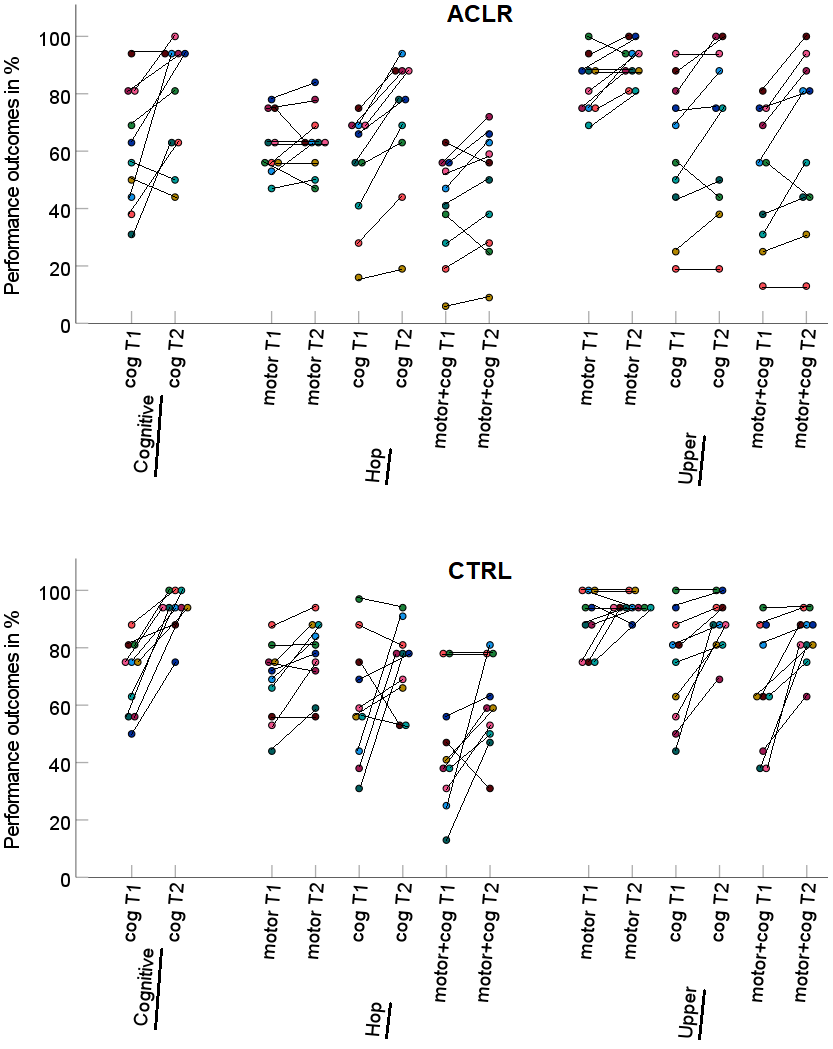


**Figure S1.** Individual paired data for the cognitive (=cog) and motor performances between tests for the cognitive test (= Cognitive), hop test (= Hop), and upper-body test (= Upper) for each group. ACLR, anterior cruciate ligament reconstruction group; CTRL, control group.
