# Supplementary material for: A Novel Dual‐Task Paradigm for Return‐to‐Sport Screening After ACL Injury: A Pilot Study
Source: Transl Sports Med. 2026 Jan 9;2026:1073180. doi: 10.1155/tsm2/1073180 (PMC12788982; doi:10.1155/tsm2/1073180)
Supplement: Supplementary file 2 — Supporting Information 2 Supporting 2: Figure S2. Bland–Altman plots for all outcomes. The X and Y axes have identical ranges to allow immediate visual comparisons between them. Identical values are stacked, so only the topmost dot is shown where present. [file TSM2-2026-1073180-s001.docx]

**SUPPLEMENT 2**


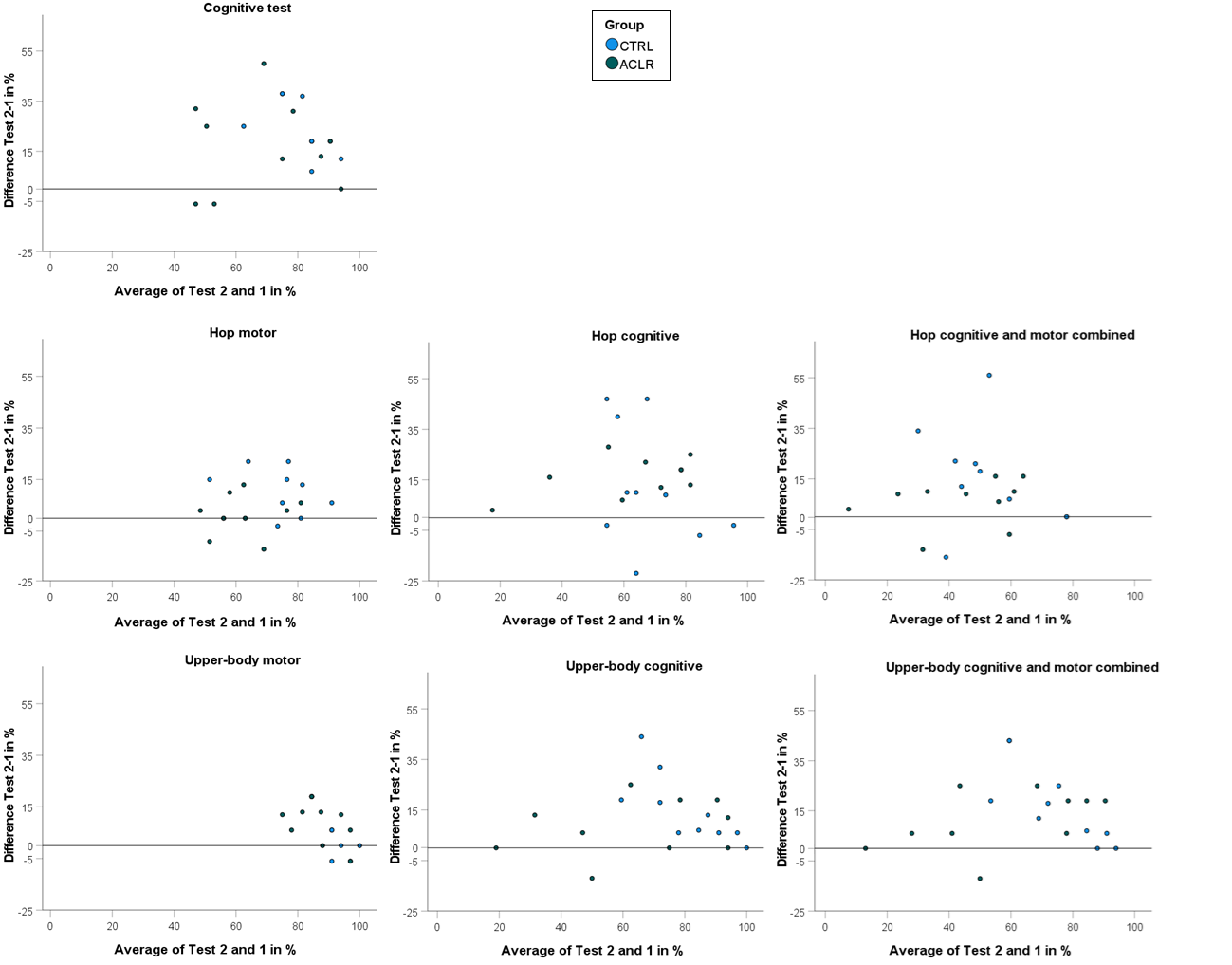


**Figure S2.** Bland-Altman plots for all outcomes. The X- and Y-axes have identical ranges to allow immediate visual comparisons between them. Identical values are stacked so only the topmost dot is shown where present.
